# App-based rehabilitation program after total knee arthroplasty: a randomized controlled trial

**DOI:** 10.1007/s00402-021-03789-0

**Published:** 2021-02-06

**Authors:** Henrik C. Bäcker, Chia H. Wu, Matthias R. G. Schulz, Thomas Sanjay Weber-Spickschen, Carsten Perka, Sebastian Hardt

**Affiliations:** 1 Department of Orthopaedic Surgery and Traumatology, Charité Berlin, University Hospital, Chariteplatz 1, 10117 Berlin, Germany; 2 Department of Orthopedics and Sports Medicine, Baylor College of Medicine Medical Center, Houston, TX USA; 3 Institute of Sports Medicine, Hannover Medical School, Podbielskistrasse 380, 30659 Hannover, Germany

**Keywords:** Total knee arthroplasty, GenuSport, Rehabilitation, Outcome, Arthritis

## Abstract

**Introduction:**

New app-based programs for postoperative rehabilitation have been developed, but no long-term study has been published to date. Thus, a prospective randomized control trial with 2-year follow-up was performed to evaluate the effectiveness of app-based rehabilitation (GenuSport) compared to a control group after total knee arthroplasty (TKA).

**Methods:**

Between April and October 2016, 60 patients were enrolled in the study. Twenty-five patients were lost to follow-up, leaving 35 patients undergoing TKA for inclusion. In this group, twenty patients received app-based exercise program and 15 were randomized to the control group. The mean age was 64.37 ± 9.32 years with a mean follow-up of 23.51 ± 1.63 months. Patients in the app group underwent an app-based knee training starting on the day of surgery; whereas, patients in the control group underwent regular physiotherapy. Functional outcome scores using the Knee Injury and Osteoarthritis Outcome Score (KOOS), Knee Society Score (KSS) and VAS of pain were analyzed.

**Results:**

In the short term, significant differences between the app group and control group in time of 10-m walk (19.66 ± 7.80 vs. 27.08 ± 15.46 s; *p* = 0.029), VAS pain at rest and activity (2.65 ± 0.82 vs. 3.57 ± 1.58, respectively 4.03 ± 1.26 vs. 5.05 ± 1.21; *p* < 0.05) were observed. In the long term, a variety of different tendencies was found, highest in KSS Function with 76.32 ± 16.49 (app group) vs. 67.67 ± 16.57 (control group) (*p* = 0.130). Additionally, patients in the app group required less painkillers (10.0% vs. 26.7%) and more likely to participate in sports (65.0% vs. 53.3%).

**Conclusions:**

An app-based knee trainer is a promising tool in improving functional outcomes such as KSS function score and VAS after TKA.

**Level of evidence:**

Level II, prospective randomized control trial.

## Introduction

Total knee arthroplasty (TKA) is indicated for treatment of end-stage osteoarthritis, to improve pain, mobility and range of motion. In long-term follow-up, excellent results with a small complication rate have been described [1].

A major aspect of achieving functional outcome is the effectiveness of rehabilitation program, in addition to having appropriate anesthesia and sound surgical techniques. This includes “enhanced recovery after surgery” (ERAS), which allows early mobilization on the same day of surgery as well as neuromuscular electrical stimulation (NMES) [2, 3]. In the first month after surgery, decrease in quadriceps muscle strength is observed in 60% of patients. Poorer stair climbing test result and slower timed 6-min walk can be observed in 90% of patients [4]. Mobile e-health app has also been shown to improve pain control and decrease opiate use in the initial postoperative period in some instances [5].

Since in-person sessions of physiotherapy are not always possible especially in the age of pandemic, a new application-based training device was invented and named GenuSport (GenuSport Knietrainer, Hannover Medical School, Germany). This application allows for isokinetic exercises that can be otherwise very costly and time intensive [6].

The GenuSport mobile app was designed to be easy to use for improving postoperative quadriceps weakness and knee motion. In the short term, functional outcome has been shown to be significantly better, although it needs additional instruction in the elderly population for proper use [7, 8]. The ease of use can improve patients’ compliance, and encourage them to perform regular exercises at home [9].

The purpose of this study was to assess the effectiveness and functional outcome of an app-based visual feedback rehabilitation program after total knee arthroplasty in a prospective randomized trial especially in long term. We hypothesized that in long term, the app-based exercise program is as good as the control group performing physiotherapy.

## Materials and methods

A prospective randomized control trial (RCT) was conducted between April and October of 2016 after obtaining internal review board approval and patient consent. All patients who underwent primary total knee arthroplasty for primary end-stage osteoarthritis were included. Patients outside the age range of 18–85 years were excluded. Patients who suffered from neurological diseases or secondary osteoarthritis, as well as those who underwent revision surgery were not included. Patients who underwent other joint replacement surgeries such as hemi-arthroplasty within 1 year prior total knee arthroplasty or missed the short or long-term exam were also excluded. For patient randomization, an independent blinded examiner prepared sealed envelopes with computer-generated randomized numbers.

A standardized protocol and pain management was developed for intraoperative and postoperative care. This included physiotherapy with active and passive knee mobilization, gait training, strengthening exercises, stair climbing, manual lymphatic drainage and cryotherapy. Exercises were performed three times daily with ice packs starting on the day of surgery for the duration of the hospital stay. Patients were allowed to walk with assistance or crutches. In addition, the app group was provided with the app-based knee trainer for 6 weeks to be used 3–5 times daily.

On postoperative day 1 and 2, therapy included continuous passive motion with a machine as well as getting out of bed and starting to walk. Simple exercises like pumping the ankles and squeezing the buttocks were included. After 1–2 weeks, patients had to focus more on gaining knee range of motion and strengthening. In addition, continuous passive motion machines were recommended in those who had less than 90° degree of knee flexion. Between the third and sixth weeks, aggressive range of motion exercises was performed with the goal of improving flexion to 100°–105° by end of week 6. Furthermore, riding of stationary bicycle and simple straight leg raise exercise were added. Neuromuscular electrical stimulation was employed to improve muscular activation of quadriceps femoris. In weeks 7 and 8, focus then turned to improving proprioception and continuing graining strength in the muscles.

Demographics such as age, gender, height, weight, BMI, and the Charlson Comorbidity Index were recorded. Before surgery as well as in the short term (within 14 days after surgery), the range of motion (ROM), circumference of the lower leg and time to walk 10 m in minutes were measured. The circumference of the lower leg was measured in three positions to an accuracy of 0.5 cm and averaged. The positions were 5 cm above the center of the patella, at the center of the patella and at 15 cm distal to the center of the patella. For functional outcome measurements, the Knee Injury and Osteoarthritis Outcome Score (KOOS), Knee Society scores (KSS) and the visual analog scale of pain at rest and at activity (R-VAS, A-VAS) were assessed in the short as well as long term (within 24 months after surgery). In addition, a clinical examination was performed before and 1 week after surgery, measuring the active and passive range of motion using a goniometer (Medigauge^®^ Digital Protractor Goniometer for Medical applications, Taylor Toolworks LLC, Columbia, USA) and time for a ten-meter walk.

**Fig. 1:**
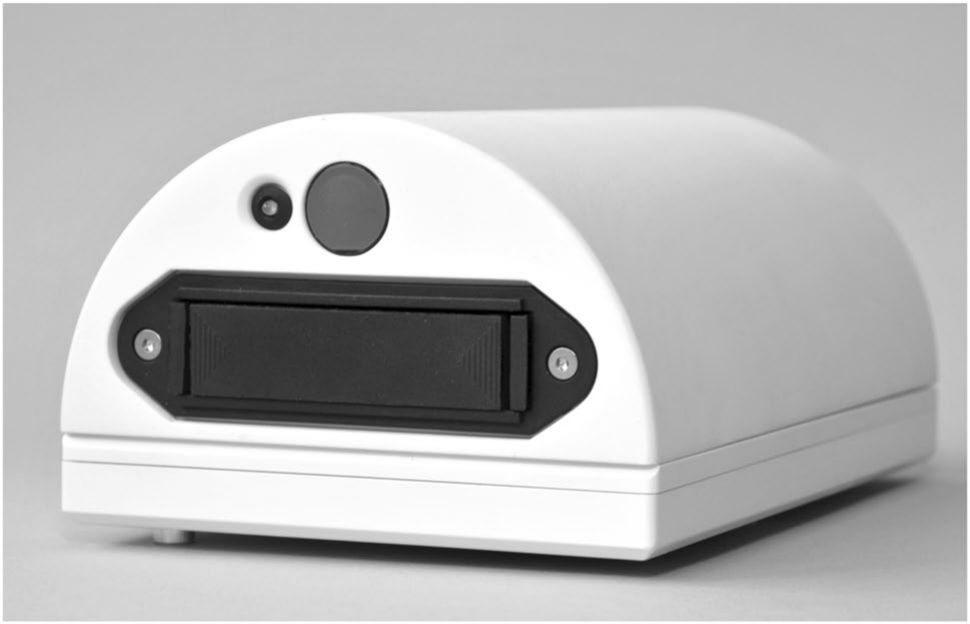
**S**ensor of the GenuSport

**Fig. 2:**
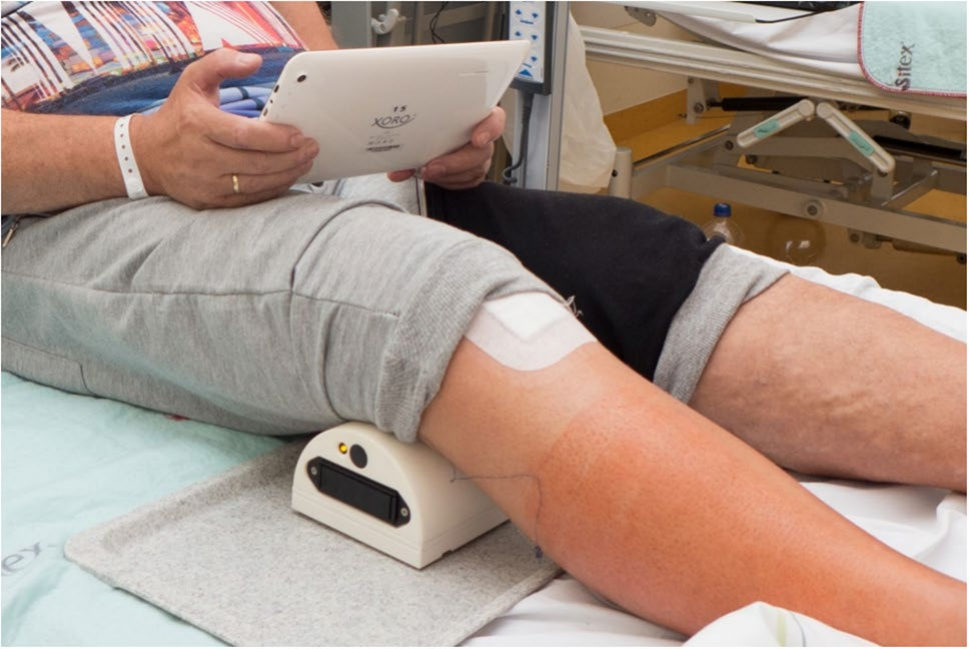
Placement of the sensor underneath the knee while holding the tablet with both hands

The app-based program consists of a knee trainer and an app called GenuSport. The knee trainer (GenuSport) contains three pressure sensors (Fig. 1) that are placed in the back of the knee while the patient is lying in a supine position with a 45° angle pillow elevating upper part of the body while holding the tablet with both hands (Fig. 2). During training, the knee is in neutral rotation without lifting the hip. The strength measured is then transmitted via Bluetooth to the app for continuous and real-time visual feedback.

**Fig. 3:**
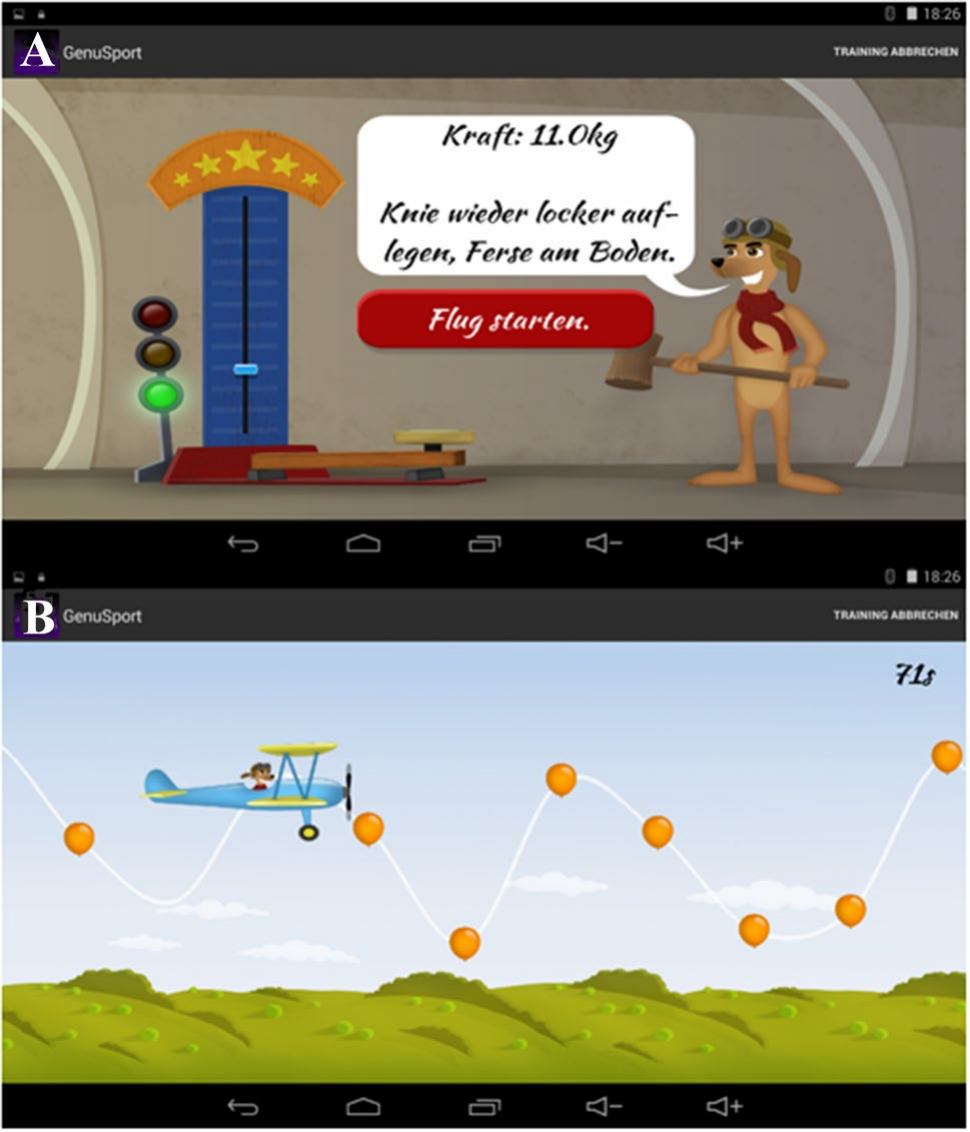
Tutorial training program; **a** “high striker game,” where the patient has to push the knee onto the sensor for 5 s; **b** “flight simulator,” where the player is supposed to keep the knee in air for 100 s

The patients can select between two different modes on the home screen. App includes a step-by-step tutorial training program that shows the history of previous training results (Fig. 2). The first mode is a game called “high striker,” where the patient has to push the knee onto the sensor for 5 s to achieve the target strength calculated by the app. The second mode is a game called “flight simulator,” where the player is supposed to keep the knee in air for 100 s (Fig. 3). At the end of each training session, patients can analyze their performance. For comparison, the other knee can be trained and tested as well. One training session lasts approximately 5 min [7].

In the short term, we focused on the active range of motion; whereas, in the long term, the functional outcome was prioritized applying the KOOS and KSS. Power analysis indicated that a difference of ten degrees in active ROM between the two groups would be detected with a sample size of 13 patients per group at 80% power. Therefore, 60 patients seemed adequate and permitted loss to follow-up. In the long term, we hypothesized that the app-based exercise program is as good as the control group in the long term. If any differences were suspected in KOOS or KSS by at least 10 points, a sample size of 14,741 patients per group was required to achieve an 80% power at a 95% confidence interval [10, 11].

For statistical analysis, IBM SPSS Statistics Version 24 (IBM Inc., Armonk, New York) and Microsoft Excel (Redmond, Washington, USA) were used. The distribution of variables was calculated by applying the Kolmogorov–Smirnov test; whereas, the independent-sample *t* test was used for normally distributed and continuous variables. The Wilcoxon rank-sum test was used for non-normally distributed variables. Furthermore, Fisher’s Exact Test was applied for nominal variables. Significances were set at the threshold of < 0.05.

## Results

**Fig. 4:**
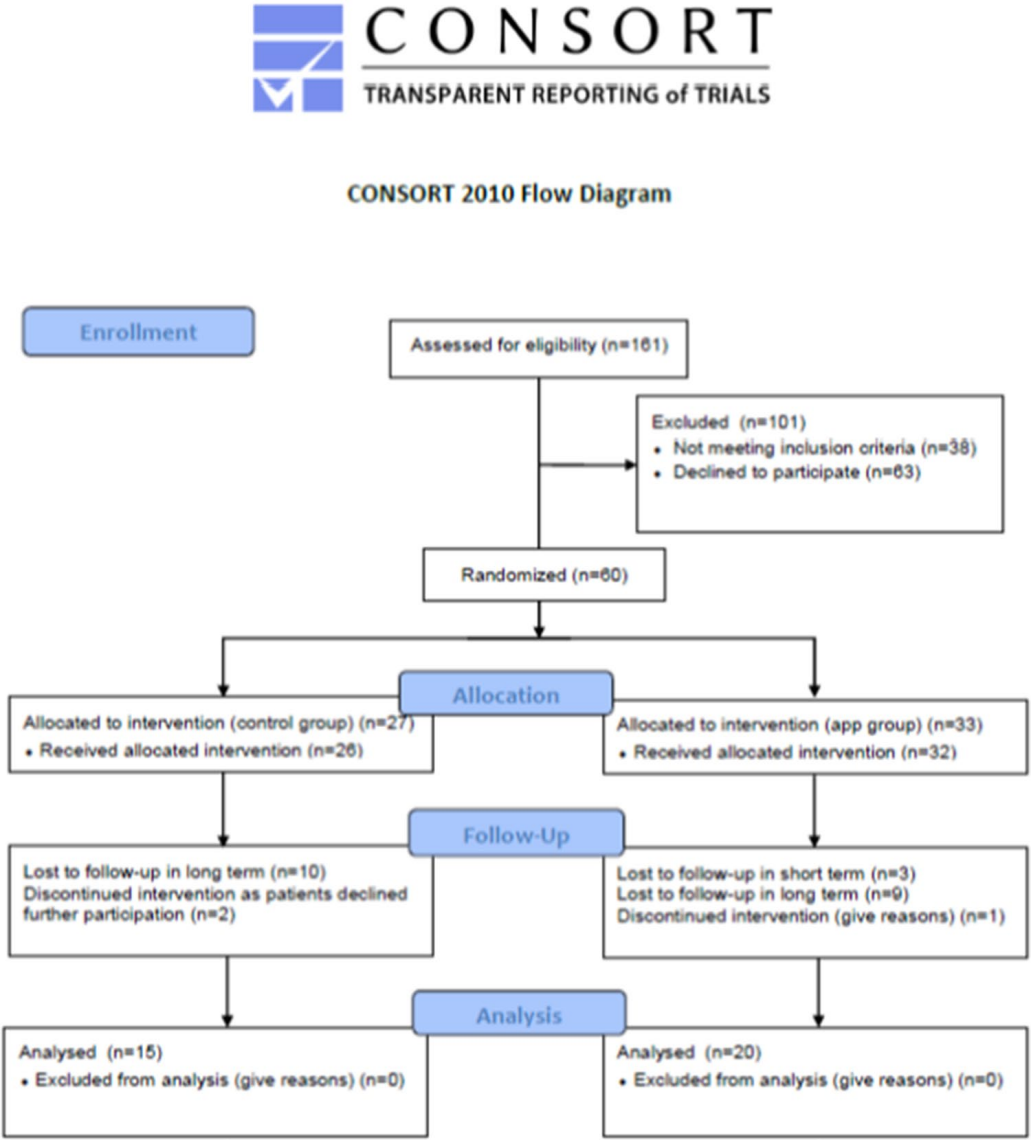
CONSOT flow chart of included patients

In total, sixty patients gave consent for inclusion in the prospective randomized trial, who were allocated into the control group (*n* = 27) and the app group (*n* = 33). Three patients declined to participate the trial after inclusion, one patient died 1 year after TKA implantation, and three patients missed short-term follow-up examination and further 18 patients missed long-term follow-up. This leaves 35 patients for inclusion in the trial (Fig. 4). No intra- or postoperative complications were observed in the cohort. Furthermore, in all patients using the app, no technical issues were reported with the app nor the trackers.

Of the 35 patients, 20 patients were randomized to the app group and the remaining 15 patients randomized to the control group. Overall, the mean age at surgery was 64.37 ± 9.32 years and female patients represent 60.0% of cases (*n* = 14/35). In this cohort, the mean height was 1.65 ± 0.09 m and the mean weight of 90.19 ± 17.81 kg (BMI: 32.95 ± 6.16 kg/m^2^). The median Charlson Comorbidity Index was 1 (range 0–5). The right knee was operated on in 54.3% of cases. The first postoperative follow-up visits occurred at 6.74 ± 0.85 days (short term) and the final follow-up examination after 23.51 ± 1.63 months (long term). No statistical significances were observed in these measurement between the two groups as shown in Table 1.

**Table 1:** Demographics, follow-up time and preoperative findings including range of motion (ROM) and circumferential

	Overall	App group	Control group	*p* value
Gender (male)	0.4 ± 0.50	0.4 ± 0.50	0.4 ± 0.51	1.000
Age (years)	64.37 ± 9.32	62.95 ± 8.25	66.27 ± 10.57	0.304
Height (m)	1.65 ± 0.09	1.66 ± 0.083	1.64 ± 0.09	0.465
Weight (kg)	90.19 ± 17.81	89.86 ± 17.96	90.64 ± 18.22	0.900
BMI (kg/m^2^)	32.95 ± 6.16	32.33 ± 5.40	33.79 ± 7.16	0.494
Charlson Comorbidity Index (median)	1 (range 0–5)	1 (range 0–5)	1 (range 0–2)	0.340
Operated knee (left side)	0.46 ± 0.51	0.55 ± 0.51	0.33 ± 0.49	0.214
Short-term follow-up (days)	6.74 ± 0.85	6.60 ± 0.75	6.96 ± 0.96	0.258
Long-term follow-up (months)	23.51 ± 1.63	23.73 ± 1.57	23.35 ± 1.65	0.505

For clinical examination, statistical significance was observed for circumference of bilateral lower extremity when comparing before and after surgery (*p* < 0.001 for the operative limb, *p* < 0.002 for the contralateral limb). A slight increase in difference was observed for the app group, which may result from the higher BMI (not significant) and more in-bed training compared to the control group. Postoperatively, the time to walk 10 m was significantly shorter within fourteen days after surgery for the app-based rehabilitation program at 19.66 ± 7.80 s vs. 27.08 ± 15.46 s for the control group (*p* = 0.029) (Table 2).

**Table 2:** Short-term clinical finding; *ROM* range of motion

	App group	Control group	*p* value
ROM active pre (°)	105.50 ± 19.71	97.50 ± 16.04	0.676
ROM active post (°)	77.15 ± 11.84	70.87 ± 12.19	0.194
ROM passive pre (°)	108.75 ± 16.39	99.57 ± 15.72	0.334
ROM passive post (°)	83.70 ± 10.42	87.33 ± 5.30	0.420
Circumferential operated leg pre (cm)	41.86 ± 3.28	49.32 ± 5.65	**< 0.001**
Circumferential operated leg post (cm)	46.66 ± 4.29	49.00 ± 7.13	0.226
Circumferential contralateral leg pre (cm)	41.41 ± 2.91	48.45 ± 6.43	**0.002**
Circumferential contralateral leg post (cm)	41.26 ± 3.89	45.20 ± 8.54	0.065
Time of 10 m walk (s) pre	11.77 ± 3.17	12.39 ± 3.04	0.837
Time of 10 m walk (s) post	19.66 ± 7.80	27.08 ± 15.46	**0.029**

Significances (*p* < 0.05) are presented in bold

For functional outcome, significant differences were observed for KOOS when comparing the app group against control group. In the short term (within 14 days after surgery), visual analog scale at rest as well as at activity was significantly better in the app group (*p* = 0.033, respectively *p* = 0.021) versus the control. In all other cases, no differences were observed. Although no significant differences were found long term, a positive trend was observed in Knee Society score. Function (KSS) was 76.32 ± 16.49 for the app group as compared to 67.67 ± 16.57 in the control group. In addition, fewer patients required painkillers in the app group (10.0 versus 26.7%, *p* = 0.207) and more patients participated in sports (65.0% versus 53.3%, *p* = 0.502) in the app group. All findings are presented in Table 3.

**Table 3:** Functional outcome before surgery, in the short (ST) as well as long term (LT); Knee Injury and Osteoarthritis Outcome Score (KOOS), Knee Society score (KSS), Visual Analog Scale at rest (R-VAS), respectively, at activity (A-VAS), Activities of Daily Living (ADL), Quality of life (QOL)

	App group	Control group	*p* value
KOOS pain pre	42.40 ± 13.32	37.84 ± 12.54	0.373
KOOS pain ST	55.99 ± 12.32	53.21 ± 11.26	0.586
KOOS pain LT	81.73 ± 21.44	80.37 ± 15.34	0.793
KOOS symptoms pre	50.62 ± 13.80	48.57 ± 17.33	**0.024**
KOOS symptoms ST	61.65 ± 13.93	63.08 ± 9.13	0.773
KOOS symptoms LT	64.47 ± 16.56	61.55 ± 14.58	0.561
KOOS ADL pre	45.84 ± 12.84	40.78 ± 12.52	0.385
KOOS ADL ST	54.58 ± 15.06	50.98 ± 9.80	0.491
KOOS ADL LT	77.17 ± 21.78	77.06 ± 15.31	0.984
KOOS sport pre	15.89 ± 13.89	9.33 ± 10.83	0.986
KOOS sport ST	9.49 ± 10.43	8.06 ± 8.09	0.817
KOOS sport LT	48.55 ± 26.05	47.33 ± 28.02	0.845
KOOS Qol pre	18.36 ± 9.61	14.17 ± 12.38	0.583
KOOS Qol ST	26.72 ± 10.2	30.79 ± 10.01	0.446
KOOS Qol LT	68.42 ± 22.68	67.92 ± 22.52	0.925
KSS function pre	53.00 ± 18.8	47.67 ± 20.95	0.988
KSS function ST	43.27 ± 12.38	42.60 ± 10.35	0.906
KSS function LT	76.32 ± 16.49	67.67 ± 16.57	0.130
R-VAS pre	3.58 ± 2.21	4.28 ± 2.37	0.689
R-VAS ST	2.65 ± 0.82	3.57 ± 1.58	**0.033**
R-VAS LT	0.88 ± 1.19	0.93 ± 1.02	0.924
A-VAS pre	7.64 ± 1.21	6.70 ± 2.42	0.933
A-VAS ST	4.03 ± 1.26	5.05 ± 1.21	**0.021**
A-VAS LT	2.67 ± 2.62	2.80 ± 2.44	0.979
Painkiller intake LT	10.0%	26.7%	0.207
Sport performance LT	65.0%	53.3%	0.502

Significances (*p* < 0.05) are presented in bold

## Discussion

Technology in medicine is evolving quickly. Postoperative rehabilitation is important for a good functional outcome. This study shows that one can expect significant improvements an app-based rehabilitation program as observed in our study. For the app group compared to the control group, timed 10-m walk (19.66 ± 7.80 s vs. 27.08 s ± 15.46; *p* = 0.029), VAS pain at rest (2.65 ± 0.82 vs. 3.57 ± 1.58), and VA pain during activity (4.03 ± 1.26 vs. 5.05 ± 1.21; *p* < 0.05) showed encouraging results, respectively. Although no significances were observed long term, a variety of trends were observed. The KSS function was 76.32 ± 16.49 for the app group compared with 67.67 ± 16.57 in the control group (*p* = 0.130). Furthermore, patients in the app group required less painkillers (10.0% vs. 26.7%) and were more likely to participate in sports (65.0% vs. 53.3%).

Besides different postoperative rehabilitation protocol, body mass index, choice of surgical technique, and implementation of an outpatient total joints program may all have major impact on functional outcome. Biomechanically, a dynamic flexion contracture during gait can be observed in painful TKAs (KOOS with a cut-off at 6/20) as well as a slight valgus alignment (− 1.5°) during stance and slight internal rotation of the combined components (− 1.4°, SD 7.0°). This can lead to increase patellofemoral forces resulting in stiff knee gait and painful TKA. Additionally, the mean BMI was significantly higher in the painful group at an average of 31.6 kg/m^2^ compared to 28.3 kg/m^2^ (*p* < 0.05). Planckaert reported pre-rotation deformity that is sometimes under-appreciated can be another source of pain [12].

The impact of body weight was investigated by Boyce et al., who reported a significantly higher revision rate due to infection, ranging from 14 to 32% in the morbidly obese group versus 0 to 25% in the non-obese group. Additionally, significantly lower functional scores before and after surgery in KSOS and KSFS were noted for the non-obese group, although the mean difference in improvement remained the same in KSOS and comparable for the KSFS. Nevertheless, all patients showed comparable improvements in knee function [13].

When looking for the operative procedure, patella resurfacing TKA has been shown to be a good alternative to the standard TKA without. Significant improvements in the rate of anterior knee pain and reoperation with 11.2%, respectively 17.4% were described. Likewise, for outcome scores, the KSS pain, KSS clinical, KSS functional and KSS overall were superior for the resurfacing group. However, the range of motion remains superior in the patella retaining group in TKA [14].

Functional outcome critically depends on the rehabilitation protocol, as evidenced by Fransen et al. The authors used a fast-track protocol involving general anesthesia, subvastus approach, no tourniquet use, and application of pain pumps. Immediate loading of the joint was encouraged and only short acting opiates were allowed. They found this protocol produced longer surgical time, higher blood loss during surgery (*p* < 0.001), and lower VAS scores in the first few days without any significances in the long term [15].

By 2021, there will be an estimated 3.8 billion smart phone users worldwide, rendering app-based rehabilitation quite accessible [16]. Apps can show different exercises or incorporate connected sensors as a feedback tool. As our society continues to age and become more mobile, rising numbers of total knee and hip arthroplasty patients may demand more flexibility in rehabilitation, especially in the age of pandemic [17]. Although different apps have been described, none has been studied in a randomized control trial to our knowledge.

The app-based knee trainer used for this study consists of three piezo-resistive force sensors and an electric circuit board to enable isokinetic exercises. The accuracy of forces measured by the device was validated against servo-hydraulic material testing system and force-measuring platform, revealing no significant differences. The difference in force measured between the knee trainer and the servo-hydraulic material testing system was described to be 0.63 N (0.4%) and − 0.11 N (0.7%) for the force-measuring platform. The app can continuously register forces, although some issues in time consistency have been reported [18, 19].

In our cohort, KSS function in the app group was 76.32 ± 16.49 versus 67.67 ± 16.57 in the control group (*p* = 0.130). This may explains why fewer patients required painkillers in the app group (10.0% versus 26.7%). For KOOS score, no statistical significance was observed between groups. In the short term, the novelty of the new technology may temporarily boost patient participation and therefore explain the significant differences in VAS scores at rest and at activity. Therefore, patients were likely to start training with more intensity on the day of surgery.

Early initiation of rehabilitation is essential for good outcome after total knee arthroplasty [4, 20]. Some authors have suggested that passive motion does not have any significant clinical impact on active knee flexion, pain or function [21]. Alternatives include high intensity training [22] or elastic bands, which are available in different resistances. It is important to exercise all major muscle groups, especially quadriceps [4, 23]. While elderly patients may require more detailed instructions with app-based rehabilitation, the device has the potential to increase compliance and lower costs of treatment, especially for patients who live in remote locations. Choosing the best method to participate in therapy should be a decision jointly made with patients [24, 25]. Furthermore, the application is designed as a game, which may reduce pain in a way comparable to listening to music after knee arthroplasty [8, 26].

While the benefit of preoperative body weight reduction is well known, there is still some debate whether patellar should always be resurfaced as part of TKA although the results are promising. In addition, the fast-track protocol should be applied selectively, and has the potential to improve perioperative care as evidenced by Fransen et al. This can decrease blood loss and length of surgery. Furthermore, an app-based rehabilitation intensifies the postoperative rehabilitation and early initiation and, therefore, improves outcome.

Our study has several limitations. We only included 60 patients in our study because we have a limited number of knee trainer prototypes at that time. Although it is a prospective randomized control trial, 41.7% of patients (*n* = 25/60) were lost to follow-up, leaving 35 patients for inclusion. This represented a loss of 44.4% (*n* = 12/27) in the control group and a loss of 39.4% in the app group (*n* = 13/33), leaving an imbalance in the cohort. While that is not ideal, we are able to include enough patients to surpass the minimum of 13 per group as indicated by our power analysis. According to the literature, loss to follow-up between 50 and 80% has been suggested to be acceptable by different authors [27, 28]. Our examiners were not consistently blinded, which may lead to some bias in clinical examination. However, this should not influence patient reported outcome like KOOS, KSS, R-VAS and A-VAS scores. We acknowledge it is difficult to assess acceptance of the app by patients and patient compliance in self-training.

## Conclusion

App-based postoperative rehabilitation has only been investigated in the short-term previously. We describe a prospective randomized controlled trial with a 2-year follow-up, evaluating the effectiveness of this novel way to deliver therapy after total knee arthroplasty. Our results indicate that patients with app-based trainer report pain score at rest and pain at activity that is comparable to in-person therapy, in addition to similar functional scores reported above. Allowing patients to train independently based on a playful visual feedback program may be a promising and preferred alternative in the age of pandemic.
